# Can research on entactogens contribute to a deeper understanding of human sexuality?

**DOI:** 10.1007/s43440-023-00552-7

**Published:** 2023-11-08

**Authors:** Justyna Holka-Pokorska

**Affiliations:** Department of Pharmacology and Physiology of the Central Nervous System, Institute of Psychiatry and Neurology, Sobieskiego 9, 02-957 Warsaw, Poland

**Keywords:** Entactogens, Sexuality, 3,4-methylenedioxymethamphetamine, Psychedelic-assisted psychotherapy

## Abstract

In recent years, scientific research into the therapeutic potential of psychedelic compounds has experienced a resurgence of interest. New studies have shown promising results, supporting the use of psychedelic drugs in treating various psychiatric disorders, including treatment-resistant depression, post-traumatic stress disorder, and even alcohol addiction. The FDA has recognized 3,4-methylenedioxymethamphetamine (MDMA) as a breakthrough therapy to treat symptoms of post-traumatic stress disorder. At the same time, interviews with recreational MDMA users have documented experiences of emotional intimacy while using MDMA, often without the desire for penetrative sex. However, some people have reported that MDMA increases their sexual arousal and specifically use it to enhance their sexual performance. This study aims to analyze current and planned research on the psychophysiological effects of entactogens on human sexuality. With their prosocial potential, the pharmacokinetic and neuroendocrine effects of entactogens may recreate the subjective experience of emotional intimacy, the initiation of intimate relationships, or even feelings of ‘falling in love’ with previously neutral individuals while under the influence of entactogens. This includes MDMA-induced sexual arousal-like effects observed through subjective behavioral perceptions of desire and arousal and specific physiological markers such as oxytocin and prolactin. Modern MDMA-assisted psychotherapy (MDMA-AP) protocols are transparent and follow strict ethical guidelines. However, despite these proposed ethical principles, little consideration has been given to the potential neurobehavioral effects of entactogens on the sexuality of participants in MDMA-AP protocols. The psychophysiological and sexual effects of entactogens should be discussed more openly in current MDMA-AP protocols, including the potential experience of the phenomenon of sexualized pharmacotransference.

## Introduction

The field of psychedelic research is currently experiencing a remarkable renaissance. After decades of restrictive regulations and societal stigma, scientific investigations into the therapeutic effects of psychedelics have resumed, unveiling a promising new direction for mental health treatment.

Studies on the effects of entactogens on humans are few and very scattered, especially regarding the impact of these substances on human sexuality. On the other hand, entactogens, especially 3,4-methylenedioxymethamphetamine (MDMA) also called ecstasy, have been used since the 1970s for recreational purposes and sexual enhancement but also for augmentation of the psychotherapeutic process [1].

The history of MDMA’s use in psychotherapy is intertwined with its dissemination as a recreational drug and then as an important element of ‘rave’ culture [2]. Its popularization as a street drug and its removal from legal therapeutic methods at the stage of the early development of psychedelic-assisted psychotherapy inhibited research into protocols using MDMA and other psychedelics.

At the current stage of research and development of therapeutic protocols related to the application of psychedelics, researchers and clinicians are challenged with a paradigm shift regarding the place of psychedelics in psychiatry. Psychedelic-assisted psychotherapy is being offered for symptoms of post-traumatic stress, depression, anxiety, and substance abuse [3, 4], which requires mental health professionals unfamiliar with the therapeutic potential of psychedelics in their academic training to adopt an entirely new perspective on the physiological effects of substances previously used as ‘mind-altering narcotics.’

In the case of psychotherapy augmented with entactogens (MDMA), it is particularly demanding to adopt a new therapeutic perspective regarding the substance formerly used recreationally for sexual enhancement. This issue is further complicated by the fact that modern protocols for research on entactogens largely dissociate their assumptions from earlier pro-sexual uses of entactogens and do not address issues related to assessing sexual parameters among the studied variables (Table 1). At the current stage of the development of studies on MDMA, there are no planned studies listed in the International Register of Clinical Trials that would be specifically dedicated to exploring the sexual effects of MDMA.

**Table 1:** Registered Phase 1 or Early Phase 1 clinical trials concerning the socio-emotional effects of MDMA in healthy individuals

*N*	Randomized controlled studies of MDMA on healthy participants	Primary outcome measures	Secondary outcome measures	Sexuality measure
1	Acute effects of 3,4-methylenedioxymethamphetamine (MDMA) with and without a booster dose NCT05809271 Sponsor: University Hospital, Basel, Switzerland Phase 1, Interventional Estimated enrollment: *N* = 24 Recruiting since 01.06.2023	Subjective effect duration for ‘any drug effect’ assessed by visual analog scale (VAS)	Subacute effects on general and mental well-being (WEMWBS, GHQ-12, SPANE) Subacute effects on subjective sleep quality, Effects on life satisfaction, well-being, and appreciation before and after study (BFW/E, GLS,) Effects moderation by personality traits (NEO-FFI, FPI-R, SPF, HEXACO, DSQ-40) Plasma concentrations of MDMA, MDMA- metabolites, Plasma concentrations and plasma levels of oxytocin	No
2	Effects of MDMA-like Substances in Healthy Subjects (MDMA-like) NCT04847206 Sponsor University Hospital, Basel, Switzerland Phase 1, Interventional Estimated enrollment: *N* = 24 Recruiting since 01.10.2022	Subjective Effects (5 Dimensions of Altered States of Consciousness 5D-ASC, Stimulation on the Visual Analog Scales (VAS) assessing the intensity and duration of the stimulant effects	Mood (BDI, SCL-90-R, Adjective Mood Rating Scale -AMRS, List of complaints LC), Emergence and intensity of phenomenon occurring in altered states of consciousness (State of Consciousness Questionnaire, Spiritual Realms Questionnaire), Psychological Insight (Psychological Insight Questionnaire) Personality Measures (NEO-FFI, Freiburger Personality Inventory -FPI-R, Saarbrücker Personality Questionnaire -SPF, HEXACO Personality Inventory, Defense Style Questionnaire DSQ-40) Plasma levels of oxytocin, vasopressin, prolactin, cortisol, S-MDMA, R-MDMA. S-MDA, R-MDA	No
3	Acute Effects of 2C-B Compared with MDMA and Psilocybin in Healthy Subjects NCT05523401 Sponsor: University Hospital, Basel, Switzerland Enrollment: *N* = 24 Time frame: 18 months Duration: 06.2023–06.2024	Acute subjective effects––5 dimensions of altered states of consciousness	Acute Subjective Effects––Visual Analog Scale (VAS) repeatedly used to assess subjective alterations in consciousness, The Adjective Mood Rating Scale (AMRS) Autonomic effects Plasma levels of 2C-B, MDMA, and psilocybin, oxytocin, BDNF, State of Consciousness Questionnaire, Spiritual Realms Questionnaire), Psychological Insight (Psychological Insight Questionnaire) Personality Measures (NEO-FFI, Freiburger Personality Inventory -FPI-R, Saarbrücker Personality Questionnaire -SPF, HEXACO Personality Inventory, Defense Style Questionnaire DSQ-40)	No
4	Effects of MDMA-like Substances in Healthy Subjects (MDMA-like) NCT04847206 Sponsor: University Hospital, Basel, Switzerland Phase 1, Interventional Estimated enrollment *N* = 24 Recruiting since 23.12.2021	Acute subjective effects—Visual Analog Scales (VAS) assessing the intensity and duration of the stimulant effects, Plasma levels of MDMA, MDA	Personality measures NEO-FFI, Freiburger Personality Inventory -FPI-R, Saarbrücker Personality Questionnaire -SPF, HEXACO Personality Inventory, Defense Style Questionnaire DSQ-40 Emotion Processing (Multifaceted Empathy Test- MET, Face Emotion Recognition Task-FERT), Autonomic Effects	No
5	The Role of Personal Experience for the Therapeutic Attitude in the Context of Substance-assisted Therapy Training NCT05570708 Sponsor: University Hospital, Basel, Switzerland Interventional, Other Estimated enrollment: *N* = 45 Duration: 10.2022- 06.2029	The primary endpoint is changed on the TASC-2 scale of the Therapeutic attitude questionnaire (ThAT)	Semi-structural interview Therapeutic attitude questionnaire (ThAT) Compassion with self-SOC-SS Compassion with Others- SPCS-O Empathy Questionnaire—SPF/IRI 5 dimensions of altered states of Consciousness Questionnaire 5D-ASC Challenging experience questionnaire -CEQ Acceptance /avoidance-promoting experiences questionnaire APEQ Psychological Insights Questionnaire- PIQ Connectedness scale—CSOWD Emotional breakthrough inventory -EDI Berner Subjective Well-Being Questionnaire BFW/E Persisting effects questionnaire PEQ	No
6	Prosocial Effects of MDMA (PEM) NCT05948683 Estimated enrollment: *N* = 34, Interventional, Early Phase 1 Sponsor: University of Chicago Recruiting since 21.07.2023–06.2024	Natural Language Processing using large language model—Differences in speech content across all conditions Facial expression analysis using HUAWEI software––Changes in emotional expressions during conversations	Oxytocin- changes in oxytocin levels across all conditions, Self-reported feelings of connection using Likert scale conversation questionnaires for ratings of connectedness Affective touch- Ratings of pleasantness, intensity and want more touch (1–7) after differing velocities of touch	No/Yes
7	Drug Effects on Interpersonal Interaction (DEI) NCT05123716 Estimated enrollment *N* = 30, Time frame: 72 h Interventional, Early Phase 1, Pair partner design Sponsor: University of Chicago Duration: 2021.08.01–2022-05-01	Profile of Mood States––measures individuals’ mood states	The POMS measures individual positive and negative mood states. The POMS contains 30 items and assesses six identified mood factors: Tension-Anxiety, Depression-Ejection, Anger- Hostility, Vigor-Activity, Fatigue-Inertia, and Confusion-Bewilderment	No/Yes
8	Effects of Drugs on Responses to Brain and Emotional Processes (MAT) (Effects of MDMA on Responses to Affective Touch in Individuals With a Range of Autistic Traits compared to healthy controls) NCT04053036 Time frame: 6 weeks Interventional, Early Phase 1 Enrollment: *N* = 24 Sponsor: University of Chicago Duration: 08.08.2019–12.04.2021	Change in responses to affective touch	Participants will complete an affective touch task during which time they will rate the pleasantness of the touch	No/Yes
9	Psychological Effects of Methylenedioxymethamphetamine (MDMA) When Administered to Healthy Volunteers (MT-2) NCT04073433 Interventional, Phase 1 Sponsor: Multidisciplinary Association for Psychedelic Studies Estimated Enrollment: *N* = 150 Recruitment since 12.2023 Time frame: 9 weeks	Change from Baseline in Self-Compassion Scale (SCS) total score––a 26-item self-report measure of self-compassion 9 weeks post-enrollment	No	No
10	Effect of Methylenedioxymethamphetamine (MDMA) (Serotonin Release) on Fear Extinction (MFE) NCT03527316 Sponsor: University Hospital, Basel, Switzerland Interventional, Early Phase 1 Enrollment: *N* = 30 Duration: 18.10.2019–24.12.2020 Time frame: 12 months	Fear extinction measured by Skin conductance response to conditioned stimuli Fear extinction measured by Fear-potentiated startle to conditioned stimuli	Subjective effects measured by Visual Analog Scale, State-trait anxiety inventory state (STAI-S) Autonomic Effects Plasma Concentration MDMA, oxytocin	No
11	Effects of MDMA Co-administration on the Response to LSD in Healthy Subjects (LSD-MDMA) NCT04516902 Sponsor: University Hospital, Basel, Switzerland Interventional, Phase1 Estimated enrollment: *N* = 24 Duration: 01.01.2021–22.08.2022 Time frame: 12 months	Acute subjective effects—Visual Analog Scale (VAS) assessing the intensity and duration of the stimulant effects, 5 Dimensions of Altered States of Consciousness 5D-ASC, Autonomic effects	Emergence and intensity of phenomenons occurring in altered states of consciousness (State of Consciousness Questionnaire, Spiritual Realms Questionnaire). Effect moderation through personality traits -personality measures NEO-FFI, Freiburger Personality Inventory -FPI-R, Saarbrücker Personality Questionnaire -SPF, HEXACO Personality Inventory, Defense Style Questionnaire DSQ-40 Emotion Processing (Multifaceted Empathy Test- MET, Face Emotion Recognition Task-FERT), Autonomic Effects Plasma levels of MDMA, LSD, oxytocin, BDNF	No
12	Effects of MDMA on Emotional and Social Memories NCT03050541 Interventional, Sponsor: University of Chicago Enrollment: *N* = 84 Duration: 12.2014–09.2016 Time Frame: 90 min	Probability of Accurately Recalling Visual Stimuli	Probability of Accurately Recognizing Visual Stimuli	No
13	Study of the Effects of MDMA/Ecstasy on Water Regulation, Sleep, and Cognition. (2C) NCT01053403 Sponsor: California Pacific Medical Center Research Institute Enrollment: *N* = 12 Interventional, Phase: non-applicable Duration 04.2010–02.2011 Time frame: 1–48 h	Time course, severity, and characteristics of MDMA discontinuation in experienced MDMA users given a known dose of MDMA, Relate observed discontinuation effects to sleep data: polysomnography, wrist actigraphy, and self-report sleep measures Assess the acute effects of MDMA on water and sodium homeostasis	Document the acute effects of MDMA on self-reported measures, including positive and negative arousal, autonomy, and sociability Document the acute effects of MDMA on behavioral measures of economic decision-making Document the acute effects of MDMA on autobiographical speech and memory Measure the effects of MDMA on ADMA	No
14	Emotional Effects of Methylphenidate and MDMA in Healthy Subjects NCT01465685 Sponsor: University Hospital, Basel, Switzerland Phase 1, Interventional Time Frame: 24 h Enrollment: *N* = 16 Duration: 12.2011–01.2013	Subjective effects during 24 h––subjective effects are repetitively assessed by standardized questionnaires	Emotional and cognitive empathy Multifaceted Empathy Test (MET) and Facial Emotion Recognition Task Prosocial behavior—Social Value Orientation slide-measurement test Neuroendocrine parameters: prolactin, cortisol, epinephrine, norepinephrine, oxytocin, pro-vasopressin, vasopressin, estrogen, and progesterone MDMA plasma levels	No
15	Effects of MDMA and Methylphenidate on social cognition NCT01616407 Sponsor: University Hospital Basel, Switzerland Interventional, Early Phase 1 Enrollment: *N *= 30 Time frame: 7 h Duration: 08.2012–04.2013	Effects on social cognition (emotion recognition and empathy)	Subjective effects are repetitively assessed by standardized questionnaires Blood pressure, neuroendocrine plasma levels, drug plasma concentration, genetic polymorphism	No
16	Effects of MDMA on social and emotional processing NCT01849419 Sponsor: University of Chicago Interventional, Basic Science Time Frame:15 min Enrollment: *N* = 65 Duration: 07.2010–03.2013	Emotional Recognition (MDMA, oxytocin, placebo)	Subjective response to MDMA, oxytocin, placebo (Ratings of ‘feel drug’, ‘feel High’, ‘feel sociable’) Motivation to socialize Cardiovascular response	No
17	Effects of Methylphenidate, Modafinil, and MDMA on Emotion processing in Humans: A Pharmaco-fMRI Study NCT01951508 Sponsor: University Hospital, Basel, Switzerland Interventional, Early Phase 1 Time frame: 1,5 h Enrollment: *N* = 24 Duration: 09.2013–01.2016	Effect on amygdala and striatum BOLD signal responses to emotional stimuli Effect on amygdala and striatum BOLD signal responses to emotional stimuli	Empathy and social behavior Subjective effects: (MET, FERT, SVO) Neuroendocrine effects Physiological effects Genetic polymorphisms	No
18	Psychological Effects of Methylenedioxymethamphetamine (MDMA) When Administered to Healthy Volunteers (MT-1) NCT01404754 Sponsor: Multidisciplinary Association for Psychedelic Studies Enrollment: *N* = 107 Time frame: 24 h Interventional, Basic Science Duration: 01.2011–08.2022	Profile of Mood States (POMS)	Interpersonal closeness measure Brief Symptom Inventory (BSI) Columbia Suicide Severity Rating Scale Neuroticism Extroversion Openness Personality Inventory Columbia Suicide Severity Rating Scale Physiological Effects	No

Data obtained from ClinicalTrials.gov

Abbreviations for psychological tools used in the assessment of the socio-emotional parameters in individual research protocols introduced by the authors of the protocols. Explanations available in the ClinicalTrials.gov

The purpose of this paper was to review research on the physiological effects of entactogens on human sexual function against other physiological functions of these substances and therapeutic protocols of psychotherapy augmented with entactogens.

An additional objective of the study was to highlight the blind spots in the field of contemporary therapeutic protocols, which at the current stage of their development require thorough research on their therapeutic effects, especially in terms of the impact on human intimate and sexual relationships. An innovative element of this study is to draw attention to the risk of pharmacologically induced erotic transference or countertransference, which may occur during entactogen-enhanced psychotherapy. This phenomenon requires both further description in terms of the theoretical model and empirical research.

### Entactogens, the specific subclass of psychedelic substances

Psychedelics, also known as serotonergic hallucinogens, are potent psychoactive substances that can modify perception and mood while influencing various cognitive functions. The history of psychedelics can be traced back to a time before written records, and they were utilized by ancient cultures in a variety of socio-cultural and ritualistic settings [5].

**Fig. 1:**
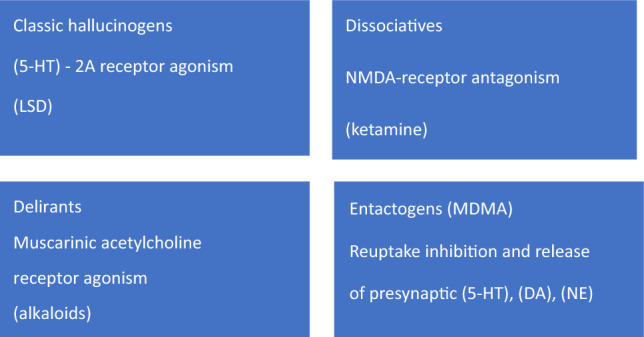
Subclasses of psychedelic substances based on [5, 6] with modification. *DA*, dopamine; *(5-HT) -2A receptors*, 5-hydroxytryptamine (serotonin) -2A receptors; *5-HT*, 5-hydroxytryptamine (serotonin); *LSD*, Lysergic *acid* diethylamide; *MDMA*, 3,4-methylenedioxymethamphetamine; *NMDA* receptors, N-methyl-D-aspartate glutamate receptors; *NE*, noradrenaline

Psychedelic substances can be categorized into several subgroups. Among them, the serotonergic hallucinogens, which primarily exert their effects by acting as agonists (or partial agonists) on brain serotonin 5-hydroxytryptamine (5-HT) 2A receptors, are considered the classic hallucinogens or psychedelics. Additionally, there are other substances that fall within the category of consciousness-altering psychedelics, including dissociatives like ketamine, deliriants such as anticholinergic drugs like atropine or scopolamine, and entactogens, with 3,4-methylenedioxymethamphetamine (MDMA) being one of the most well-known representatives [5] (as listed in Table 1). Although all of these substances induce profound alterations in consciousness, they do so through distinct mechanisms of action (as illustrated in Fig. 1, Table 2).

Entactogens (empathogens) are the subclass of psychedelic substances characterized by a special socio-emotional potential. Entactogens have been shown to produce feelings of emotional openness, oneness, empathy, or sympathy. The term entactogens was coined by David E. Nicholas in 1986 to delineate a subclass of substances ‘producing a touching within’ [6]. The most popular representative of the entactogen subclass is MDMA, next to MDA and MDE (Fig. 1).

3,4-methylenedioxymethamphetamine (MDMA, ‘ecstasy,’ ‘molly’) was synthesized for the first time in 1912. Before MDMA was classified in the US as a controlled substance it was used between 1970 and 1985 as an adjunct to psychotherapy because of its properties enhancing the feelings of emotional closeness and decreasing the defensiveness in the therapeutic process [1, 7, 8]. In the seventies and eighties, MDMA was used for its anxiolytic and prosocial effects to help patients with trauma-related disorders, anxiety disorders, and in couples therapy [9]. When it rapidly spread in the eighties as a recreational drug, it also led to its worldwide ban in 1985–86. In 1984 Drug Enforcement Administration (DEA) recommended that MDMA be classified as Schedule I of the Controlled Substances Act. After the use of MDMA was made illegal, some group of psychotherapists in the US and Europe continued to use MDMA to enhance the psychotherapeutic process.

The acceptable medical use of this substance in the US was deemed to require the US Food and Drug Administration (FDA) approval. Eventually, the first FDA-approved MDMA study was launched in 1992 [10].

The first official research on the therapeutic uses of MDMA looked at the symptoms of post-traumatic stress disorder (PTSD) in women [11] and in small group of both sexes (*N* = 20) [12]. The first study aimed to investigate the safety and preliminary efficacy of administering different doses of MDMA-assisted psychotherapy within a psychotherapeutic setting to six women suffering from chronic PTSD resulting from sexual assault [11]. This study demonstrated that low doses of MDMA, ranging between 50 and 75 mg, proved to be both psychologically and physiologically safe for all subjects involved [11]. The second study evaluated the efficacy of MDMA in treating symptoms of post-traumatic stress disorder (PTSD) in twenty patients with chronic PTSD [12]. This study revealed that MDMA was significantly more effective than a placebo in reducing PTSD symptomatology, with an 83% clinical response rate in the active treatment group compared to 25% in the placebo group. Furthermore, there were no reports of serious adverse events related to the use of MDMA [12].

Following the completion of six international phase 2 clinical trials involving 107 patients with refractory PTSD, the results were reviewed by the FDA [13–17]. In August 2017 the Multidisciplinary Association for Psychedelic Studies (MAPS) obtained from the FDA a Breakthrough Therapy Designation (BTD) status for 3,4-methylenedioxymethamphetamine (MDMA)-assisted psychotherapy for the treatment of PTSD based on the results of pooled analyses [18]. FDA has granted also the second breakthrough therapy designation for psilocybin in treatment-resistant depression (TRD) in 2018 and major depressive disorder (MDD) in 2019 [19].

Since then, interest in psychedelics and entactogens among researchers and in the entrepreneurial segment has grown exponentially.

Starting on July 1, 2023, MDMA and psilocybin can be prescribed by authorized psychiatrists in Australia. This development followed the reclassification of these two substances by the Therapeutic Goods Administration (TGA) from a schedule 9 listing, typically reserved for prohibited drugs with addictive potential only used in clinical trials or research, to a schedule 8 classification, which permits prescription under controlled conditions. However, this decision has not been without its critics who argue against the unjustified banning of psychedelics [20]. The regulatory framework imposed by the TGA mandates that only psychiatrists who have received proper training and authorization are allowed to prescribe MDMA and psilocybin. Additionally, each patient case must gain approval from a human research ethics committee, and these treatments must be administered in conjunction with psychotherapy.

## Controversies and promises of MDMA-assisted psychotherapy

Early therapists advocating for the use of psychedelic-based psychotherapy relied on their own experiences with psychedelics or different methods of inducing altered states of consciousness. An altered state of consciousness can be achieved through meditation [21], as well as through the psychopharmacological effects of psychedelic substances [22, 23]. There is a widespread consensus that psychedelics cannot be fully explained by their pharmacological properties alone [24], but rather by individual factors (referred to as ‘set’) and contextual factors (referred to as ‘setting’) that play a pivotal role in some of the observed clinical benefits of psychedelics [24, 25].

During the early research on psychedelics, no PAP therapeutic protocols were created that could be fully replicated in modern research and therapeutic procedures. Modern pioneering psychedelic research centers use their therapist training protocols where therapists are required to undergo their own psychedelic experience as part of their training [23, 26].

A training protocol for therapists participating in MAPS-sponsored MDMA randomized clinical trials was developed in 2012 by Annie and Michael Mithoefer who also disclosed that the training protocol was based on the previous psychedelic experiences of its authors [2].

Because in the 1980s the early procedures of PAP were not strictly regulated by psychotherapeutic associations, some therapists working with PAP were quite open in organizing the psychotherapeutic sessions. This has led to several cases of lack of respect for the physical boundaries of patients by therapists conducting entactogen-assisted psychotherapy sessions [27].

Since then, psychotherapeutic associations have proposed the rule of conducting sessions by two therapists of both sexes, which was supposed to result in greater control throughout the session and over the development of transference and countertransference emotions [2, 3, 12, 28].

Most modern centers that conduct the processes of psychotherapy augmented with psychedelics for scientific or clinical purposes use the psychedelic potential for generating altered states of consciousness and mystical experiences [29]. For the application of psychedelics, the centers follow a new ‘reset paradigm,’ which postulates that the mystical type ‘peak experience’ induced by psychedelics is correlated with therapeutic effects and are indicative psychological marker for the restoration of healthy network connectivity in the brain [3, 29–31].

The importance of psychotherapeutic support for individuals undergoing psychedelic-assisted treatment is a central tenet in the field of psychedelic science [23, 32, 33]. In clinical trials, this support takes the form of a structured approach, encompassing preparation sessions before dosing, therapeutic guidance during dosing, and integration sessions following dosing. Preparation sessions involve establishing a therapeutic relationship, exploring participants’ mental health concerns, and discussing the objectives to be pursued during the dosing session. This aspect of the session goals is referred to as the ‘intention for the dosing session’ in the methodology of psychedelic-assisted therapy [23]. During these preparation sessions, participants receive comprehensive information about the substance, its effects, potential risks, and benefits. Furthermore, strategies are developed to address any challenging experiences that may arise during dosing sessions. In clinical trial dosing sessions, which typically extend for 6 to 8 h, two therapists are present, adopting a ‘non-directive approach’ aimed at nurturing the participant’s ‘inner healing intelligence’ [26]. Participants are encouraged to wear eyeshades, listen to instrumental music, and focus on their internal experiences [23, 34]. The therapists maintain this ‘non-directive approach’ to support and facilitate the expression of the participants’ inner healing processes.

MDMA-assisted psychotherapy (MDMA-AP) is the structured form of psychedelic-assisted psychotherapy developed by the Multidisciplinary Association for Psychedelic Studies (MAPS). As outlined in the MDMA-AP manual, this approach operates on the assumption that the effects of the substance, including reduced fear, heightened interpersonal trust, and enhanced positive emotions toward oneself and others, create a corrective environment for re-experiencing traumatic events in the context of a compassionate and supportive therapeutic relationship [26]. MDMA-assisted psychotherapy entails the administration of single doses, closely monitored under continuous medical supervision, with sessions occurring at two to three-week intervals. Before drug administration, preparatory sessions are conducted, and these are followed by psychotherapy sessions aimed at assisting individuals in integrating therapeutic changes into their daily lives [4].

**Table 2:** Basic pharmacological information on the most popular entactogens [2, 35, 36]

Substance	MDA	MDMA	MDE
Name	3,4-Methylenedioxyamphetamine	3,4-Methylenedioxymethamphetamine	3,4-Methylenedioxyethyloamphetamine
Dosage	75–125 mg	75–140 mg	100–160 mg
Half-life time (hours)	8–10	4–6	3–5
Duration of effects (hours)	7–9	R (-) -MDMA = 6 S (-) -MDMA = 3,5	R (-) -MDE = 8 S (-) -MDE = 4
Type of effects	Entactogen/hallucinogen	Entactogen	Entactogen

An updated *group model* of psychedelic-assisted psychotherapy (PAP) has been developed, employing substances like MDMA or psilocybin [3]. As an example, within the Swiss group model of PAP, MDMA is predominantly utilized in the initial phase of the psychotherapeutic process to enhance motivation for change and bolster the therapeutic alliance. Subsequent therapeutic efforts are concentrated on improving emotional self-regulation, addressing negative self-perceptions, and mitigating structural dissociation to build tolerance for trauma exposure during the next phase of the psychotherapeutic process, which often involves exposure to LSD. This model is underpinned by the assumption that many psychological disorders can be traced back to neglect or early-life trauma, including perinatal or transgenerational experiences, resulting in developmental constraints or specific trauma-related disorders [3]. In the realm of MDMA-assisted psychotherapy, there exists a consensus among scientists and clinicians that the augmentation of psychotherapeutic methods with MDMA holds particular promise in the treatment of disorders associated with attachment insecurities. These disorders include but are not limited to PTSD, depression, anxiety disorders, obsessive–compulsive disorder, suicidality, substance use disorders, and eating disorders [3, 4]. It has even been proposed as a potential approach for victims of sexual assaults experiencing symptoms of chronic post-traumatic stress disorder [37, 38].

For the majority of therapists and psychiatrists who neither have their own experience with psychedelic substances nor participate in research on psychedelics, the very encounter with innovative methods is a great challenge and undermines the basic elements of their current paradigm of working with psychiatric patients [39]].

The philosophical change of the perception concerning the role of the psychiatrist in the PAP and especially in the MDMA-AP process applies to several elements: duration of the pharmacological treatment, the structure of psychedelic application sessions, the interpretation of the altered states of consciousness, mystical experiences and spirituality, the role of music and artistic expression, and finally the personal experience with the use of psychedelics (Table 3).

**Table 3:** Main differences between the psychedelic-assisted models and non-psychedelic models in psychiatry and psychotherapy

	Basic assumptions of the psychedelic-assisted therapeutic models	Basic assumptions of the non-psychedelic therapeutic models
1.	Therapeutic application of the substances previously used to create the laboratory models of psychoses	Most of the mind-altering substances considered in psychiatry to be narcotics
2.	Altered states of consciousness are considered a therapeutic tool	Altered states of consciousness commonly considered pathological (dissociation or prodromal symptoms of psychosis); indication for the antipsychotic pharmacological intervention
3.	The short duration of the pharmacological intervention accompanied by the sessions of psychedelic preparation and session of the integration of the psychedelic experience	Long-term treatments (pharmacological intervention of continuous treatment for several months, years, or indefinitely) Psychotherapy is usually considered as an adjunct to pharmacotherapy, non-mandatory during pharmacological treatment
4.	Detailed definition of the patient attitude and context of the psychedelic experience	Patients’ attitudes toward pharmacotherapy, context, and therapeutic process are rarely analyzed, non-obligatory
5.	Spiritual, mystical context present in the therapeutic process	Spiritual or mystical context absent in the pharmacotherapeutic process or considered as the psychotic phenomenon
6.	Music is an invaluable and essential ingredient of most forms of psychedelic-assisted psychotherapy	Music and other forms of artistic expression independent of the treatment context
7.	Experience with psychedelics required or recommended for professionals conducting psychotherapeutic processes	Experience with psychedelics is considered the harmful use of illegal substances

The researchers, participants, and universities face conflicting interests and incentives, which is strictly important in the context of avoiding missteps in the introduction of psychedelics to the set of psychiatric methods without a thorough understanding of all the effects that psychedelic substances have on humans [39].

## Completed and planned research concerning the effects of MDMA on human sexuality

To assess the state of knowledge concerning the effects of entactogens on sexual human functions, a systematic review of the literature using PubMed database has been conducted. The updated search for the scope of this mini-review was performed on the 1st of July 2023 with the following keywords (MDMA OR 3,4-methylenedioxymethamphetamine OR ecstasy) AND (‘sexuality’ OR ‘sexual response’ OR ‘intimate relationships’). The author has received 148 records most of which 143 did not meet the criteria for describing the psychophysiological effects of MDMA on human sexuality.

Given the absence of enough results in the PubMed Database author decided to search for the clinical trials registers. In the EU Clinical Trial Register using the following keywords: (entactogen OR MDMA OR methylenedioxymethamphetamine), the search did not return any results. In the database clinicaltrials.gov using the same topic, we have received 96 results.

For the scope of this mini-review, we have decided to further analyze the clinical trials concerning the neurophysiology of entactogens (MDMA) in humans considering the Phase 1 clinical trials conducted on healthy participants. The primary purpose of this analysis was the evaluation of the advancement of the research on the physiology of entactogens in terms of their impact on human sexuality. We have analyzed the primary and secondary outcomes of these trials in terms of the assessment of the effects of MDMA on sexual functions or the ability to engage in intimate relationships.

Of the 96 clinical trials registered, 17 concerned Phase 1 studies on healthy volunteers, while other studies concerned the assessment of the impact of MDMA on selected psychopathological symptoms in people with mental health problems (Table 1) [40].

Including studies listed in the register as of August 15, 2023, so far, studies with MDMA have been designed for people with symptoms of the following disorders, post-traumatic stress disorder (PTSD), combat stress disorder, mood disorder, anxiety disorder, substance-related disorders, alcohol use disorder, amphetamine-related disorders, drug addiction, social anxiety in autistic adults, anorexia nervosa restricted type, binge-eating disorder, adjustment disorders, OCD, and schizophrenia [40].

Whereas from the first phase or early first phase studies, only three studies were indirectly designed to assess the effects of MDMA on the evaluation of the preferences of the partner of the conversation under the influence of MDMA or placebo (NCT05123716) and self-reported feeling of connectedness and affective touch received from the familiar and unfamiliar person (NCT05948683).

The first study was conducted between 2021 and 2022 and was planned to evaluate the effect of drugs on personal interactions in the same-sex pair partner design. The protocol proposed the subjects’ engagement in a 45-min semi-structured conversation with a novel partner under the influence of MDMA and with another partner under the influence of a placebo. At the end of each session, participants were supposed to rate their feelings about the partner, preferences about the partner, and the evaluation of the feelings of closeness to the subject under the influence of MDMA and placebo on the test day.

The aim of the second study on the prosocial effects of MDMA was to test the hypothesis that MDMA produces greater prosocial effects when administered in the presence of a familiar, compared to an unfamiliar person. In this study, it was proposed to investigate whether the prosocial effects of MDMA are greater when interacting with a partner to whom the participant feels close and more connected, compared to an unfamiliar partner. To establish familiarity in one of the study groups, the study proposed a procedure during which two same-sex partners, who were initially strangers, were supposed to engage in a 45-min conversation with a ‘small talk’ condition or ‘deep talk’ condition that could produce feelings of connection, being understood and liking their partners (NCT05948683). The protocol of the study, planned to be conducted between 2023 to 2024, did not give any details concerning the method of assessment of the affective touch that participants of the experiment were supposed to receive from partners of a neutral or in-depth conversation.

The third study conducted between 2019 and 2021 was also designed the evaluation the change in responses to affective touch but in a group of individuals with a range of autistic traits. Participants were supposed to complete an affective touch task during which time they were asked to rate pleasantness of touch (NCT04053036) at the point after receiving 1.5 mg MDMA and 6 weeks after the drug administration.

In all three studies, the assessment of the effects of MDMA on the feeling of intimacy or sexual arousal was addressed only indirectly by evaluation of the feeling of connection, liking the partner of the conversation, or the evaluation of the affective touch. Experiments were designed in same-sex couples, possibly to avoid the risk of subjects engaging in intimate relationships or inducing experiences that might be interpreted as sexually related.

Users of MDMA (± 3,4-methylenedioxymethamphetamine, often referred to as ‘ecstasy’) commonly report experiencing unique psychological effects, including heightened empathy and increased prosocial feelings [41]. These ‘empathogenic’ effects also led to the exploration of MDMA’s potential in psychotherapy [26]. Nonetheless, these effects have yet to be comprehensively characterized through controlled studies. Several controlled, double-blind studies have been conducted to investigate the behavioral effects of MDMA and to unveil the psychological and neural processes underlying its prosocial impact [41–43]. A pivotal discovery in these studies has shown that MDMA reduces sensitivity to the detection of negative emotions conveyed through facial expressions, while simultaneously enhancing the ability to identify positive emotions [41]. It is worth noting that MDMA augments ‘empathogenic’ feelings but diminishes the accurate recognition of facial emotional signals associated with threats in others. These findings align with an increase in social approach behavior rather than genuine empathy. As posited by Bedi et al., this effect of MDMA on social cognition holds relevance not only for recreational use but also for therapeutic applications. In recreational users, the acute effects of the drug may alter social risk-taking behavior while under the influence.

On the other hand, these two socio-emotional processing alterations could facilitate both positive social interactions with strangers and interactions with a psychotherapist [41, 44] and could be important for the further development of MDMA-assisted psychotherapy protocols.

In conclusion, it should be emphasized that none of the randomized controlled studies published so far on the socio-emotional effects of MDMA, nor even any planned studies available in the register of controlled trials, assess the impact of MDMA on intimate relationships and human sexual functions.

## Reports of MDMA recreational users as a starting point for further research into the sexual effects of entactogens

MDMA is recognized among drug users for its positive social-emotional effects, such as increased feelings of empathy, interpersonal closeness, and sociability [45–47]. Furthermore, it is acknowledged as an enhancer of the sexual experiences [48]. Except for alcohol and cannabis, MDMA was the most commonly used drug during sexual activities in the group of 22,289 respondents of all sexes and sexual orientations [48]. It is particularly prevalent in the group of gay, bisexual males, and other men who have sex with men (GBMSM). Within the group of chemsex practitioners, MDMA is popular alongside other substances also used for the enhancement of sexual activity, i.e., ketamine, methamphetamine, GHB/GBL, or mephedrone [49].

Research concentrated on the evaluation of the motivations of MDMA users in naturalistic settings has shown that one of the main motivations for using this substance is sexual enjoyment [50] or sexual enhancement [51, 52]. The primary findings of the study involving young MDMA users in intimate relationships with one or more partners, living in economically disadvantaged environments affecting interpersonal relationships, indicated that both men and women primarily used MDMA for sexual pleasure. While men reported more frequent use of MDMA for sexual enjoyment, women more commonly reported using MDMA to alleviate psychological or physical discomfort, especially in relationships marked by distrust [50].

In McElrath et al.’s study, the majority of respondents had used MDMA six months before the interview, with a significant percentage having consumed the drug on 100 or more occasions [51]. Most respondents reported experiencing emotional closeness while using MDMA, albeit without a desire for penetrative sex. On the other hand, some respondents, particularly homosexual and bisexual females, had used MDMA specifically to enhance their sexual experiences. The episodes of sexual activity under the influence of MDMA were found to be associated with risky sexual behaviors, such as engaging in sex with multiple partners and not using condoms.

A study investigating the experiences of young women (aged 18–29) from New York found that MDMA use was associated with heightened both sexual aspects (e.g., desire, arousal, orgasmic intensity) and sensual aspects (e.g., deep emotions, emotional closeness, feelings of love, and affection). However, these experiences were more commonly categorized as sensual. A majority of women who engaged in sexual activity after the application of MDMA believed that the drug did not lead to risky sexual behavior. Nevertheless, some women reported engaging in sexual behaviors they might not have otherwise engaged [52].

The results of a study involving 679 nightclub attendees (ages 18–25) indicated a strong association between MDMA use and heightened perceived sexual effects, including perceived sexual attractiveness of oneself and others, sexual desire, length of intercourse, and sexual openness [53]. Furthermore, increased sensitivity of the body and sex organs, as well as heightened sexual intensity, were most closely linked with MDMA use [53]. This study revealed that MDMA had distinct sexual effects compared to marijuana and alcohol, with each substance carrying different risks and benefits for users [53]. In contrast, other studies have reported that MDMA did not increase responses to sexual stimuli, suggesting that the effects of MDMA are not necessarily linked to increased sexual attraction [54]. Some users have also reported that MDMA did not enhance sexual desire or enjoyment but had a positive impact on emotional closeness and a greater willingness to engage in sexual activity [51, 55, 56].

In the study conducted by Zemishlany, the effects of MDMA on various aspects of sexual response were assessed in a small group consisting of 35 healthy recreational MDMA users (20 men and 15 women aged 21–48) [56]. The study focused on four major domains of sexual activity: desire, erection (lubrication in women), orgasm, and satisfaction. It was found that MDMA moderately to profoundly increased desire and satisfaction in over 90% of the subjects. Orgasms were delayed but perceived as more intense. However, erection was impaired in 40% of the men [56]. These findings suggest that while MDMA enhances sexual desire and the perception of greater satisfaction, it can also impair sexual performance.

There are also descriptive reports from small groups of adult couples who have used MDMA for relationship enhancement [57]. These couples have described positive effects on communication, and intimate bonding, and referred to it as a way to provide a ‘relationship tune-up,’ among other lasting changes to their relationships.

## New protocols of MDMA-assisted psychotherapy and the risk of ‘sexualized pharmacotransference’ processes

The protocols of psychedelic-assisted psychotherapy are transparent and strongly relate to the ethical rules characterizing most of the therapeutic processes of various schools of psychotherapy. The ethical guidance of psychedelic-assisted psychotherapy introduced strict rules of no physical contact with participants during the session, strict respect of sexual boundaries, and examining the sexual countertransference by the therapists [58]. Although ethical emphasis relies on ‘respect the sexual identities and expression of participants and validate participants’ processes that might relate to sexuality and sexual healing,’ little is known about the potential for precipitating the intimate or sexual effects of entactogens both outside and inside of the setting of MDMA-assisted psychotherapy. The interactions of psychotherapists and patients in the process of psychotherapy can induce transference and countertransference feelings in both participants of the interaction regardless of the neutrality and transparency of the whole process. Transference is described as the unconscious projection of feelings onto another significant individual. Sigmund Freud first introduced the concept of transference, which has since been explored within various psychoanalytic perspectives, including object relations, Kleinian, Jungian, and Lacanian thought [59]. Transference can be seen as a way the brain makes sense of current experiences by overlaying them with past ones, potentially limiting the reception of new information. Transference can manifest in various forms, including positive, negative, or sexualized transference, the latter encompassing erotic or eroticized transference. Erotic transference is considered a mature form of sexualized transference in which the patient may experience sexual fantasies toward the psychotherapist while being aware that these fantasies are unrealistic. In contrast, eroticized transference represents an intense and irrational erotic preoccupation with the psychotherapist, often accompanied by overt demands for love or sexual engagement [60]. Sexualized transference can potentially have detrimental consequences, depending on how it is managed within the therapeutic process [60, 61]. Each major psychoanalytic approach has developed its methods for preventing the development of erotic transference or harnessing it for therapeutic benefit [59]. Conversely, some short-term therapeutic approaches may not emphasize the risk of erotic transference, preassuming that the brief nature of the process inherently guards against sexualized transference or that it naturally resolves itself.

The protocols for psychedelic-assisted psychotherapy can be considered short-term therapeutic approaches that include a limited number of preparatory and integrative sessions.

In the protocol for MDMA-assisted psychotherapy used in clinical trials conducted by the Multidisciplinary Association for Psychedelic Studies, it is a requirement to have two psychotherapists present, one of each gender, during the preparatory and integrative sessions (as outlined in the MAPS Code of Ethics) [58]. It can be presumed that the span of 2–3 preparation sessions may not be sufficient to establish a deep emotional connection with the therapist, and there may be limited risk of the development of transference feelings toward the psychotherapist. In this model, the presence of two therapists during the administration of entactogens in a medical context primarily serves to ensure the patient’s safety while they navigate various sensory aspects of the altered state of consciousness.

On the other hand, during integrative sessions, the patients are expected to process the effects of the psychedelic experience with the therapist. The therapist’s neutral behavior during these sessions might be interpreted as friendly or even as bearing signs of closeness and intimacy, especially under the influence of previous MDMA use. The prosocial effects of entactogens, which enhance feelings of emotional closeness and connection, have been preliminarily described in the studies conducted thus far. Hence, it appears particularly important for MDMA-Assisted Psychotherapy (MDMA-AP) therapeutic protocols to include psychoeducational elements that can prepare participants for the possibility of sensory experiences that they may interpret as related to intimacy or sexual experiences with unclear meanings. Therapeutic protocols should also guide how therapists should conduct themselves during integrative sessions if patients wish to better understand any sexual sensory experiences that may have arisen during the application of entactogens or other psychedelics. 

Since the socio-emotional effects of MDMA are associated with feelings of connection, oneness, and even intimate bonding, the application of MDMA could induce a specific subcategory of transference feelings, i.e., eroticized transference that could be called *the pharmacotransference process*.

The risks of the development of sexual, eroticized transference during the application of MDMA in clinical settings were not sufficiently addressed at the current stage of work on these protocols. Little is known about methods of facilitating the resolution of the possible sexual ‘pharmacotransference’ during MDMA-AT sessions.

According to Thorsten Passie, the behavioral effects of entactogens resemble experiences that mimic the physiology of the sexual response cycle along with the phases of excitement, plateau, orgasm-like culmination, and reflection. The phenomenological features of the psychological state induced by MDMA, in turn, show some similarities with features of the post-orgasmic state. In addition, MDMA also induces a prominent increase of prolactin plasma levels with a similar time kinetic compared to the post-orgasmic prolactin increase [62]. More recent studies evaluating the pharmaco-physiology of MDMA application in humans have revealed that the physiological effects of MDMA also include the release of oxytocin [63]. This effect is not seen with classic hallucinogens like LSD or stimulants [64]. The findings of MDMA as a potent oxytocinergic further strengthen the validity of hypotheses comparing the oxytocin-related physiology of the human sexual response [65] and the parasexual effects of MDMA as comparable to processes underlying human bonding and intimate relationships.

From the point of view of evolutionary psychology, human sexual response with orgasmic culmination is understood as a powerful stimulus that through positive reinforcement may increase the chances that the copulation will occur again with the same partner. The effect of orgasm on the development and shaping of partner preferences may involve catalysis of the neurochemical mechanisms of bonding [66]. Partner-related cues experienced in the presence of sexual reward come to elicit a representation of that reward and thereby become desired features that identify the partner as the beloved. This process is especially potent during an individual’s first sexual experience but may be additive throughout the lifespan, such that several love maps develop [67, 68].

At the current stage of research on entactogens, we cannot rule out that these substances affect changes in the perception of other people’s behavior as intimate behavior or modify the subject’s behavior into sexually motivated behavior. Therefore, during the further development of the psychotherapeutic methods based on entactogens, it is worth considering the possible risk of inducing eroticized ‘*pharmacotransference*’ and ‘*pharmacocountertransference*’ regarding both participants of the MDMA-AT process, i.e., patient and psychotherapist [69, 70]. At this point in the development of the protocols, the main postulated method to avoid the development of sexualized transference is sessions conducted by two therapists of both genders.

Based on naturalistic reports of MDMA-related physiological effects, some aspects of MDMA application experience could be interpreted as related to intimacy or sexuality by sensitive or ‘hyper-sensitive’ patients.

High sensitivity to the application of entactogens may apply in particular to women who did not have former contact with MDMA. In the study of Liechti et al. concerning 74 subjects (54 male, 20 female) with no prior contact with MDMA, the psychoactive effects of MDMA (75–150 mg) were more intense in women than in men. In women, MDMA-induced perceptual changes, thought disturbances, and fear of loss of body control were more frequent and intense [71].

Therefore, the aspects related to the sexual dimorphism of the response to entactogens and the psychosexual physiology that accompanies their application should be taken into account in further work on the development of MDMA-AP protocols.

## Discussion and future perspectives

Amid the contemporary resurgence of psychedelics as potential medicinal tools for addressing various conditions, the narrative surrounding MDMA has transformed. It has shifted from being perceived as a perilous street drug to a groundbreaking therapy in the realm of mental health. Nevertheless, the tale of MDMA remains somewhat incomplete, often framed within a binary discourse that pits its recreational misuse against its psychotherapeutic and medical applications [72]. Despite significant advancements in utilizing mind-altering substances to treat psychiatric disorders, there are gaps in our comprehensive understanding of the psychological mechanisms through which these substances affect mood, behavior, and human sexuality.

As presented in Table 3, when comparing classical and psychedelic-based models of psychiatric care, Psychedelic-Assisted Psychotherapy (PAP) and MDMA-Assisted Psychotherapy (MDMA-AP) represent entirely novel medical technologies situated within the context of the psychedelic experience, drawing on sensory modalities previously unexplored in the field of psychiatry. As depicted in Table 1, in clinical studies aimed at deepening our understanding of the psychophysiology of entactogens, some research initiatives are underway, focusing on aspects such as prosocial effects or responses to sensory stimuli. These include evaluations of connection with conversation partners and assessments of the pleasantness of affective touch, as seen in studies like NCT05948683, and changes in response to affective touch, as observed in NCT04053036. However, it is noteworthy that within the largest contemporary registry of clinical trials [40], there are currently no planned studies investigating the effects of entactogens on intimacy and sexual response. As an increasing number of countries legalize the therapeutic use of entactogens, it is essential to recognize that there remains a gap in our understanding of the psychophysiological aspects of their actions. This gap pertains to areas such as potential transference emotions concerning the person of psychotherapists or the risk of ‘sexualized pharmacotransference’ that may accompany even short-term therapeutic interactions like in PAP, even if these aspects are not the primary focus of short-term therapeutic approaches.

Controlled laboratory studies, employing well-defined psychological constructs, play a pivotal role in unraveling how these substances yield their therapeutic benefits. However, it is crucial to acknowledge that there exist significant methodological disparities between clinical studies aimed at investigating therapeutic outcomes and laboratory studies designed to probe the underlying processes that may account for these therapeutic effects [57].

In this mini-review, we have tried to examine the basic assumptions concerning the story of the therapeutic use of MDMA, the circumstances of its popularization as a street drug, and its removal from legal therapeutic methods at the stage of the early development of psychedelic-assisted psychotherapy. We have also outlined the challenges faced by clinicians who want to continue working on the development of MDMA protocols, both in individual in group settings.

We have revealed that at the current stage of the development of studies on MDMA, there are no planned studies listed in the International Register of Clinical Trials that would be specifically dedicated to exploring the sexual effects of MDMA.

Based on naturalistic reports of MDMA-related physiological effects, many aspects of clinical MDMA application need better understanding and in-depth research in controlled settings.

Understanding human sexual behavior requires continued research and a balanced approach to the simplicity of the definitions of sexual response with normal and abnormal arousal, desire, performance, and orgasm with the array of exceptions to those definitions that characterize sexual function and dysfunction in real people [73]. According to Pfaus et al., many questions concerning human sexual functions are ‘obscured by research review committees, with certain moral limitations imposed by ethics review boards and government agencies.’ There is much that ‘cannot be done, either because of ethical concern, or the lack of sufficient technology.’

When designing research into the social-emotional effects of entactogens, the philosophical underpinnings of researching human sexuality and the complicated history of these substances doubly complicate the process of conducting these studies. The foundational assumptions surrounding the narrative of MDMA’s therapeutic usage, its journey from being a street drug to its exclusion from legal therapeutic practices, have hindered the evolution of psychedelic-assisted psychotherapy methods in their early stages. On the contrary, current efforts to employ MDMA within clinical contexts for the treatment of post-traumatic stress disorder and to expand its potential applications to other psychiatric conditions have made significant progress [74, 75]. Nevertheless, there is a need for a more comprehensive understanding and extensive research into various aspects of clinical MDMA applications, particularly regarding its physiological effects, including those on sexuality, as evident from naturalistic reports.

Many clinical trials involving new psychiatric drugs utilize the Arizona Sexual Experience Scale (ASEX) to evaluate sexual function [76]. This five-item rating scale measures sex drive, arousal, vaginal lubrication/penile erection, the ability to reach orgasm, and satisfaction from orgasm. Nevertheless, when it comes to research assessing the effects of psychedelics, it might be beneficial to develop a new tool capable of evaluating psychological aspects related to sexuality more comprehensively. In the long-term follow-up of patients who have taken part in psychedelic-assisted psychotherapy protocols, it could be valuable to assess any changes in their sexual activity preferences or inclinations toward risky sexual behavior.

The challenges of translating findings from recreational and street drug use to clinical settings are demanding but essential. This calls for a blend of rigorous laboratory and clinical research that bridges the scientific methodology with the insights derived from ‘street discoveries’ regarding the effects of entactogens on human sexuality. On the flip side, this unique opportunity for further in-depth research in a scientific setting on a substance previously used extensively for recreational purposes and studied in naturalistic contexts should not be missed.

Neglecting topics related to the assessment of the effects of entactogens on intimate relationships and sexuality in planned or future clinical research on entactogens might have unforeseen consequences for patients participating in entactogen-assisted therapy protocols. One of the risks highlighted in this review pertains to the potential development of ‘pharmacotransference,’ a topic not yet explored in the scientific literature within this context. Conducting randomized trials that incorporate entactogens with a substance-based intervention augmented by psychotherapeutic intervention demands meticulous attention during study design and result interpretation. The widespread adoption of psychotherapeutic methods enhanced with entactogens for specific psychiatric disorders necessitates extensive research into the psychophysiological and sexual effects of these substances, drawing insights from studies conducted on healthy volunteers.

## Data Availability

Data sharing is not applicable to this article as no datasets were generated or analyzed during the current study.

## Abbreviations

2 C-B: 4-Bromo-2,5-dimethoxyphenethylamine)
FDA: The US Food and Drug Administration
5HT-2A receptor: Serotonin 5-hydroxytryptamine (5-HT) 2A receptor
MAPS: Multidisciplinary Association for Psychedelic Studies
MDA: 3,4-Methylenedioxyamphetamine
MDE: 3,4-Methylenedioxyetyloamphetamine
MDMA: 3,4-Methylenedioxymethamphetamine
MDMA-AP: Methylenedioxymethamphetamine-assisted psychotherapy.
LSD: Lysergic acid diethylamide
PAP: Psychedelic-assisted psychotherapy
PTSD: Post-traumatic stress disorder
TGA: Therapeutic Goods Administration
TRD: Treatment-resistant depression
